# The feasibility of enhanced pore space utilization in CO_2_ storage reservoirs using an artificially emplaced Si-gel flow barrier

**DOI:** 10.1038/s41598-023-36349-0

**Published:** 2023-06-08

**Authors:** Teya Cossins, Achyut Mishra, Ralf R. Haese

**Affiliations:** 1 School of Geography, Earth and Atmospheric Sciences, University of Melbourne, Melbourne, VIC 3053 Australia; 2 The Peter Cook Centre for Carbon Capture and Storage Research, University of Melbourne, Melbourne, VIC 3053 Australia; 3 Present Address: WSP Australia Pty Limited, Level 11, 567 Collins Street, Melbourne, VIC 3000 Australia

**Keywords:** Hydrology, Geochemistry

## Abstract

Carbon capture and storage is a key technology to abate CO_2_ emissions. One of the challenges towards ensuring the efficiency and the security of CO_2_ storage in reservoirs, such as open saline aquifers, is the low pore space utilization. This study investigates the feasibility of using an artificial Si-gel barrier to enhance pore space utilisation in such reservoirs under variable geological conditions. Conceptually, enhanced CO_2_ capillary trapping is achieved by emplacing a disk-shaped, low-permeability barrier above the CO_2_ injection point forcing the injected CO_2_ to migrate laterally underneath the barrier before transitioning to buoyancy-controlled migration. Multiphase fluid flow simulations were conducted to test the feasibility of this concept. Sensitivity analysis revealed that the barrier exhibits a strong control on CO_2_ plume geometry. Specifically, the relative impact of the barrier diameter on increasing the CO_2_ plume width, reducing the plume height and enhancing trapping varied between 67 and 86%. Capillary trapping was enhanced by 40–60% with a 20 m increase in barrier diameter in low permeability reservoirs. Additionally, the results indicate that the barrier can enhance the security of trapping CO_2_ in high permeability reservoirs. Results were tested for the South-West Hub reservoir, a case study area in Western Australia.

## Introduction

Geological carbon sequestration is a crucial transitional technology for mitigating anthropogenic CO_2_ emissions from the utilization of fossil fuel resources^1,2^. Conventional CO_2_ storage reservoirs have a caprock or a structural trap^2,3^. On the other hand, open saline aquifers without a defined caprock are often referred to as unconventional CO_2_ storage reservoirs where the primary CO_2_ trapping mechanism is capillary trapping rather than structural trapping^4^. In case of capillary trapping, disconnected globules of CO_2_ are permanently trapped within the pore space^5^.

Conventional reservoirs are primary targets for CO_2_ sequestration as they offer storage capacities on gigaton scale which is required for meeting CO_2_ emission targets^6^. However, such ideal storage reservoirs may not be available within a reasonable distance to CO_2_ emission sources. Additional infrastructure and time required to transport the captured CO_2_ to conventional storage reservoirs could significantly increase the cost of the operation making it economically unviable. Hence, there is an increasing interest in utilising unconventional reservoirs for geological CO_2_ sequestration. A key real-world example in this context is the Svalbard pilot-scale CCS project where off-shore geological CO_2_ storage was found to be too expensive to develop^7^. This motivated the development of CO_2_ storage opportunities in the locally available onshore sedimentary reservoirs which lacked a well-defined structural trap. A storage capacity of as much as 50 million tons has been estimated for the unconventional reservoirs in Svalbard which has been deemed to be sufficient to mitigate local CO_2_ emissions^7^. The other key open aquifers for unconventional CO_2_ storage around the globe include the Mey Sandstone Member (UK)^8^, Heimdal Sandstone Member (UK)^8^, Maureen Formation (UK)^8^, Bunter Sandstone (UK)^9^, Mount Simon Sandstone (USA)^9^ and Basal Cambrian Sandstone (Canada and USA)^9^ with each estimated to have a net storage capacity at gigaton scale, provided adequate procedures are in place to avoid excess pressure build-up^9^.

Effective pore space utilisation is critical to ensure the security of stored CO_2_ as well as maximizing the overall storage capacity in storage reservoirs^10^. The development of technologies to enhance pore space utilisation is key to the feasibility of sequestration projects where a conventional gigatonne scale storage capacity is not practicable. The storage efficiency in homogeneous open aquifers can be as low as 2–5% compared to a trapping efficiency of as high as 70–80% for depleted oil and gas fields comprising caprock and lithological heterogeneity^10^. Several technologies have been proposed to improve the storage efficiency such as the use of foams^11^, gels^12^ and biomineralization^13,14^. The use of a synthetically created silica-gel barrier is a novel technology which can potentially enhance storage capacity and security for homogeneous open aquifers.

**Figure 1:**
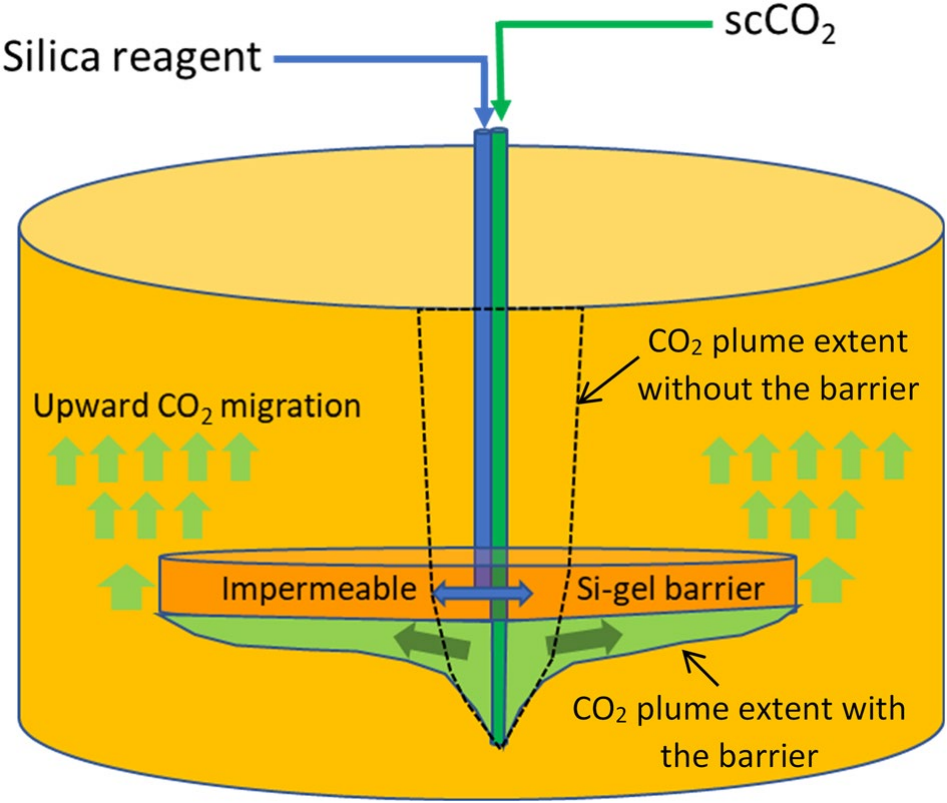
Schematic representation of the CO_2_ plume without a barrier (stippled line) and with an impermeable Si-gel barrier (green arrows). The width of the CO_2_ plume and, thus, the capillary trapping capacity is expected to be larger with a Si-gel barrier.

The gel is a polymerised form of amorphous silica which forms through the reaction of an alkaline sodium silicate solution and an acid^15^ or from an alkaline sodium silicate solution alone with an adjusted pH so that gel formation occurs within hours to days^16,17^. The precipitation of the gel clogs the pore space, thereby reducing porosity and permeability of the reservoir which blocks CO_2_ flow pathways^18–20^. The concept of a silica gel barrier in the context of carbon sequestration arose in response to the need for mitigation and remediation technologies to ensure the long-term security of CO_2_^12,21^. Sodium silicate was proposed due to its durability in acidic conditions characteristic of CO_2_ storage reservoirs and the ability to form a gel. Core flood experiments at both ambient and reservoir conditions as well as numerical modelling highlighted Si-gel as an ideal material for applications in CCS^22–24^_._ More recently, the concept of emplacing a Si-gel flow barrier above an injection point was proposed as a technology to increase the lateral migration of CO_2_, thereby enhancing the storage capacity^25^ (Fig. 1)_._ The in-situ formation of the gel can be achieved by co-injecting an acid and a Na-Si reagent^25,26^. The reactions between the two result in the precipitation of the polymerised form of amorphous silica which fills the pore space. A previous study^25^ suggested the following three steps lead to the formation of the Si-gel effectively: (1) Injection of the acid for 30 h, (2) Injection the reagent for 70 h, (3) Co-injection of both the solutions for 20 h. This strategy results in the formation of the gel in about 5 days^25^. Post precipitation, the barrier undergoes syneresis which is a process where the gel loses water contributing to the enhanced structural strength of the barrier^27^. Previous experimental studies have suggested that the barrier can have a structural strength of about 600 bar/m for shallow reservoir conditions^28,29^. This strength is about 1000 times higher than the capillary entry pressure of commonly encountered sedimentary rocks of similar sizes, such as mudstone intraformational baffles, in siliciclastic CO_2_ storage reservoirs^30^. Highly concentrated silica gel solution could have 20 times the viscosity of water under CO_2_ storage conditions^31^. Additionally, the material required for gel formation are relatively low cost and have minimum environmental impact^25^.

These factors make silica gel advantageous over other technologies aimed at enhancing CO_2_ storage capacity such as cements, geopolymers, foams and chemical reactive barrier. Cements pose the risk of degradation under acidic conditions of CO_2_ storage^32^ while geopolymers can be difficult to handle^33^. The efficiency of foams depends on bubble size which is a function of formation pressure^34^ and it is difficult to create a uniform distribution of a chemically reactive barrier in the reservoir due to heterogeneity in mineral precipitation rates^18,19^. The existing research suggests that the emplacement and efficiency of the silica gel barrier is not limited by any such factors.

While previous studies^25,26^ have focussed on optimum conditions for the formation of the flow barrier, its performance towards improving trapping efficiency under variable geological conditions has not been investigated. This study aims to explore the impact of an artificially implemented barrier on enhanced pore space utilization and CO_2_ trapping security for a range of unconventional reservoir types, especially the homogeneous open aquifers. The following two questions were addressed:

A) Under what geological conditions is the Si-gel flow barrier most effective?
B) How much can the CO_2_ trapping efficiency and security increase by using the synthetic barrier?

## Methodology

### Reservoir model scenarios

A total of 49 reservoir models were generated with variable reservoir properties and Si-gel flow barrier sizes. These models can be grouped into three categories: 2D models with an impermeable barrier, 2D models with a semi-permeable barrier and 3D models representing a potential CO_2_ storage site. The 2D models were designed to explore the effectiveness of the Si-gel barrier under different geological conditions. Hence, the properties in the 2D models were varied synthetically to capture a range of barrier and reservoir properties. The 3D simulations were aimed at testing the performance of the barrier under realistic reservoir conditions with industry standard injection rates. The properties in the 3D models were also synthetically varied but they were based on the values recorded for the SW Hub, which is a potential CO_2_ storage reservoir in Western Australia^35^. The models were constructed in the PetraSim software.

A total of 30 2D geostatic models with impermeable fluid flow barriers were constructed with a domain size of 100 m × 100 m (Fig. S1). Finer grid cells were concentrated in the centre of the model to provide higher resolution near the injection cells and in proximity to the flow barrier while the vertical grid cell size was fixed at 1 m. The models comprised two rock types: reservoir rock and the barrier.

An additional 3 2D models were constructed to test the potential of semipermeable and partial barriers in improving the reservoir pore space utilisation. The first scenario captured the case where barrier permeability was similar to reservoir permeability. Such a barrier can be a result of poor-quality polymerization of the Si-gel under field conditions. The other two scenarios represented barriers with gaps which were 5 m and 15 m apart in the two models, respectively. Such partial barriers can result under field conditions using an injection sequence of water and alkaline silica solution, allowing the barrier to form as a central disk around the well, followed by a gap and then an outer ring. In the 2D models, this would appear as a segmented barrier with two evenly spaced gaps occupied with reservoir rock. The domain, grid cell and barrier dimensions were the same as in the 2D impermeable barrier scenarios.

A total of 16 3D models were built to test the barrier performance under field conditions. The domain size of the 3D models had a height of 260 m and a length of 200 m in each of the x and the y directions. The lateral grid dimensions were refined close to the injection well and the barrier. Vertical model resolution was 4 m, while the cross-sectional area of cells ranged between 6.25 m^2^ and 25 m^2^ (Fig. S2c,d).

### Static rock properties

Porosity and permeability values of reservoir rock were varied across the 30 2D models with impermeable barriers (Table S1). The values followed a normal distribution and were generated using the Monte Carlo method with their mean and standard deviation values shown in Table S2. Permeability anisotropy and the barrier diameter were randomly varied (Table S2). Our previous study has suggested that the behaviour of Si-gel flow barrier towards restricting CO_2_ migration is similar to a fine-grained lithology such as mudstone or siltstone which can be up to 50 m wide^25^. Hence, the properties of the barrier were modelled after the properties typical of such rock types. This was done to save on the computational costs required for running detailed reactive transport simulations for capturing the complete formation of the barrier in all the scenarios. This has already been shown in our previous publication^25^. The properties of the barrier remained constant throughout the scenarios, with a porosity of 0.05 and a permeability of 1 mD (Fig. S1).

For the 2D models with semi-permeable barrier, the barrier material was set to 200 mD permeability and 0.15 porosity. The other two scenarios captured a partial barrier and had the same barrier properties as the impermeable barrier cases. A mean reservoir porosity and permeability of 0.3 and 1000 mD, respectively, were used to generate the grid cell property values using the Monte Carlo method.

Porosity and permeability values in the 3D models were based on the existing reservoir model of the SW Hub^35^. These values were taken from the lower mid-section of the available Petrel model of the site centred around the GSWA Harvey 1 well, representing an injection depth of around 2000 m^35^. At this depth, the SW Hub would be considered a tight reservoir. Porosity in this section ranged from 0.06 to 0.18 (Fig. S2a) and permeability lies between 4 and 368 mD (Fig. S2b–d), with an overall average of 71 mD. Porosity followed a normal distribution (Fig. S2a) while the permeability displayed a log-normal right skewed distribution (Fig. S2b). These values were implemented as in one of the 16 3D scenarios which is, henceforth, referred to as the reference case (Fig. S2c,d). An anisotropy value, representing the ratio of vertical to horizontal permeability, of 0.7 was used in the reference case. The properties in the other 3D scenarios were approximated based on our observation of the SW Hub model as relevant sub-sections could not be extracted from Petrel due to technical limitations. The second scenario had a reservoir rock permeability 1.4 times greater than the permeability of the reference case and was constructed to capture the rock property values in other sections of the SW Hub model at similar depths. The third and the fourth cases implemented an anisotropy value of 0.1 and 0.8, respectively, representing sections of the SW Hub model at similar depths with high and low anisotropy, respectively. The next set of 4 cases used the same values as in the first 4 cases described above, except for reservoir permeability, which was 10 times greater than the value of the 4 cases above. These cases represent the sections of the SW Hub model at shallower depths of 1300–1400 m capturing different ranges of permeability and anisotropy^35^. The flow of CO_2_ in these 8 cases was simulated twice, with and without the barrier, to quantify the impact of emplacing a Si-gel barrier under realistic reservoir conditions. In the former cases, the barrier was implemented 12 m above the injection location with constant porosity and permeability of 0.05 and 1 mD, respectively while the diameter of the barrier was fixed at 39.67 m at its widest point. This is the closest approximation of a 20 m radius disc shaped Si-gel barrier implemented with the rectangular mesh used in these models (Fig. S2d).

### Dynamic rock properties

For the 2D models, relative permeability curves were implemented using a hysteretic form of the Van Genuchten model^36–38^ using the following formulation:

1 $${\mathrm{k}}_{\mathrm{r,w}}={{\mathrm{s}}_{\mathrm{e,w}}}^{1/2}{\left[1-\left(1-\frac{{\mathrm{s}}_{\mathrm{e,nwt}}}{1-{\mathrm{s}}_{\mathrm{e,wtp}}}\right){\left(1-{\left({\mathrm{s}}_{\mathrm{e,w}}+{\mathrm{s}}_{\mathrm{e,nwt}}\right)}^{1/{\mathrm{m}}_{\mathrm{r}}}\right)}^{{\mathrm{m}}_{\mathrm{r}}}-\left(\frac{{\mathrm{s}}_{\mathrm{e,nwt}}}{1-{\mathrm{s}}_{\mathrm{e,wtp}}}\right){\left(1-{\mathrm{s}}_{\mathrm{e,wtp}}^{1/{\mathrm{m}}_{\mathrm{r}}}\right)}^{{\mathrm{m}}_{\mathrm{r}}}\right]}^{2}$$

2 $${\mathrm{k}}_{\mathrm{r},\mathrm{nw}}={\left(1-{\mathrm{s}}_{\mathrm{e},\mathrm{w}}-{\mathrm{s}}_{\mathrm{e},\mathrm{nwt}}\right)}^{1/3}{\left(1-{\left({\mathrm{s}}_{\mathrm{e},\mathrm{w}}+{\mathrm{s}}_{\mathrm{e},\mathrm{nwt}}\right)}^{1/{\mathrm{m}}_{\mathrm{r}}}\right)}^{{\mathrm{m}}_{\mathrm{r}}}$$

where ${\mathrm{k}}_{\mathrm{r},\mathrm{w}}$ is the relative permeability of the wetting (water) phase, ${\mathrm{k}}_{\mathrm{r},\mathrm{nw}}$ is the relative permeability of the non-wetting (CO_2_) phase, m_r_ is the relative permeability empirical parameter taken equal for drainage and imbibition, ${\mathrm{s}}_{\mathrm{e},\mathrm{w}}$ is the effective wetting phase saturation, ${\mathrm{s}}_{\mathrm{e},\mathrm{nwt}}$ is the effective trapped saturation of the non-wetting phase, and ${\mathrm{s}}_{\mathrm{e},\mathrm{wtp}}$ is the effective turning point saturation of the wetting phase.

The effective saturations can be obtained from the following relations:

3 $${\mathrm{s}}_{\mathrm{e},\mathrm{w}}=\frac{{\mathrm{s}}_{\mathrm{w}}-{\mathrm{s}}_{\mathrm{w},\mathrm{irr}}}{1-{\mathrm{s}}_{\mathrm{w},\mathrm{irr}}}$$

4 $${\mathrm{s}}_{\mathrm{e},\mathrm{wtp}}=\frac{{\mathrm{s}}_{\mathrm{w},\mathrm{tp}}-{\mathrm{s}}_{\mathrm{w},\mathrm{irr}}}{1-{\mathrm{s}}_{\mathrm{w},\mathrm{irr}}}$$

5 $${\mathrm{s}}_{\mathrm{e},\mathrm{nwt}}=\frac{{\mathrm{s}}_{\mathrm{nw},\mathrm{r}}\left({\mathrm{s}}_{\mathrm{w}}-{\mathrm{s}}_{\mathrm{w},\mathrm{tp}}\right)}{\left(1-{\mathrm{s}}_{\mathrm{w},\mathrm{irr}}\right)\left(1-{\mathrm{s}}_{\mathrm{w},\mathrm{tp}}-{\mathrm{s}}_{\mathrm{nw},\mathrm{r}}\right)}$$

where ${\mathrm{s}}_{\mathrm{w}}$ is the wetting phase saturation, ${\mathrm{s}}_{\mathrm{w},\mathrm{irr}}$ is the irreducible wetting phase saturation, ${\mathrm{s}}_{\mathrm{w},\mathrm{tp}}$ is the turning point saturation of the wetting phase, and ${\mathrm{s}}_{\mathrm{nw},\mathrm{r}}$ is the residual non-wetting phase saturation.s_w_ and s_w,tp_ are dynamically determined over the period of simulation. s_nw,r_ is a function of turning point saturation of the wetting phase at the end of drainage and is determined using the Land’s trapping model^39^ given below:

6 $${\mathrm{s}}_{\mathrm{nw},\mathrm{r}}= \frac{1-{\mathrm{s}}_{\mathrm{w},\mathrm{tp}}}{1+\left[\frac{1}{{\mathrm{s}}_{\mathrm{nw},\mathrm{max}}} - \frac{1}{\left(1-{\mathrm{s}}_{\mathrm{w},\mathrm{irr}}\right)}\right]\left(1-{\mathrm{s}}_{\mathrm{w},\mathrm{tp}}\right)}$$

where s_nw,max_ is the maximum possible saturation of the non-wetting phase. The residual non-wetting phase saturation, s_nw,r_, is determined post the drainage-imbibition cycle in a given grid cell. As CO_2_ invades a grid cell under drainage, the saturation of CO_2_ increases until the turning point saturation is achieved. Post drainage, CO_2_ saturation value decreases under imbibition until its relative permeability becomes zero. At this point, residual CO_2_ saturation is achieved in the grid cell which is a function of the turning point saturation value. These grid cells are expected to be located behind the tailing end of the plume. Ignoring the effects of CO_2_ dissolution, the residual CO_2_ saturation is constant in these cells even when the front of the plume might still be migrating.

The modified form of Van Genuchten capillary pressure function^36–38^ has been used to account for hysteresis:

7 $${\mathrm{P}}_{\mathrm{c}}= {\mathrm{P}}_{\mathrm{o}}{\left[{\left(\frac{{\mathrm{s}}_{\mathrm{w}}-{\mathrm{s}}_{\mathrm{w},\mathrm{irr}}}{1-{\mathrm{s}}_{\mathrm{w},\mathrm{irr}}-{\mathrm{s}}_{\mathrm{nw},\mathrm{r}}}\right)}^{-\frac{1}{{\mathrm{m}}_{\mathrm{c}}}}-1\right]}^{\left(1-{\mathrm{m}}_{\mathrm{c}}\right)}$$

where P_c_ is the capillary pressure (Pa), P_o_ is the capillary strength parameter for scaling the capillary pressure curve (Pa), m_c_ is the capillary pressure empirical parameter, taken the same as the relative permeability curve.

The rock property values used in the above equations are given in Table S3. For the flow barrier, s_nw,max_ and s_w,irr_ values typical for a siltstone were taken from the literature^40^ while for the reservoir rock, these values were taken from the SW Hub model^35^.

For the 3D models, relative permeability was implemented as Corey curve^41^ based on the available data^35^ using the following functional form:

8 $${\mathrm{k}}_{\mathrm{r},\mathrm{w}}= {\mathrm{s}}_{\mathrm{ce},\mathrm{w}}^{4}$$

9 $${\mathrm{k}}_{\mathrm{r},\mathrm{nw}}= {\left(1-{\mathrm{s}}_{\mathrm{ce},\mathrm{w}}\right)}^{2}(1-{\mathrm{s}}_{\mathrm{ce},\mathrm{w}}^{2})$$

where,

10 $${\mathrm{s}}_{\mathrm{ce},\mathrm{w}}=\frac{{\mathrm{s}}_{\mathrm{w}}-{\mathrm{s}}_{\mathrm{w},\mathrm{irr}}}{1-{\mathrm{s}}_{\mathrm{w},\mathrm{irr}}-{\mathrm{s}}_{\mathrm{nw},\mathrm{max}}}$$

The Corey capillary pressure curve could not be directly implemented in TOUGH3, hence, its parameters were used to generate an equivalent Van Genuchten capillary pressure function (Eq. 7). The rock property values used in the saturation function formulations for the 3D models are given in Table S3. To save computational time for the 3D simulations, hysteresis was applied post-model run using a python script where the turning point saturations obtained from simulations were used in the Land’s trapping model^39^ to obtain the trapped CO_2_ saturation (Eq. 6).

### Numerical simulation set-up

Multiphase fluid flow simulations were performed in TOUGH3 using the ECO2N module designed for carbon sequestration in saline reservoirs^42,43^. Supercritical CO_2_ was injected under reservoir pressure temperature conditions of 150 bars and 60 °C representative of the conditions at the SW Hub^35^. Under these conditions, CO_2_ density^2^ is 686.12 kg m^−3^. For the 2D scenarios, injection occurred from the central grid cell in the basal layer of the models continuously for 5 days with an injection rate of 17,280 tonnes per annum (tpa). The plume was then observed for a further 95 days, resulting in a total simulation time of 100 days. For the 3D scenarios, the same injection location and duration was used. However, the implemented injection rate was 157,680 tpa which was based on the net rate of 300,000 tpa achieved through 9 injection wells as reported for the entire site^35^. This gives confidence in ensuring that the results are representative of field conditions even with the limited spatial and temporal scales used in simulations. Constant pressure condition was implemented in the top and side boundaries to ensure that these boundaries are open to fluid flow.

The numerical simulations are set-up under the following assumptions:

- Fluid flow rate dependency of relative permeability and capillary pressure saturation functions is not considered.
- The outer boundary of the barrier is implemented using structured grid cells assuming the impact on fluid flow around the edges of the barrier is small. An unstructured grid would be more suitable to resolve the disc shape of the barrier.
- The barrier material has been approximated with a rock type of equivalent petrophysical and flow properties rather than using polymerised form of amorphous silica which is the actual material.
- The impact of CO_2_ dissolution and mineralization on the net trapping amount has not been considered in this study.

### Calculation of the impact of the barrier on CO_2_ plume geometry and trapping

The height and the width of the stabilised plumes were measured from the simulation results. Plume height was taken to be the maximum distance between the injection point and the shallowest footprint of the plume. Similarly, plume width was taken to be the maximum lateral distance between the plume boundaries which was generally observed directly underneath the barrier.

In order to quantify and compare the amount of CO_2_ trapped via capillary trapping across all the scenarios with varying barrier diameters, the amount of CO_2_ stored per meter of plume height (C_PMH_) was calculated for each realization using the following equation:

11 $${\mathrm{C}}_{\mathrm{PMH}}= \frac{{\mathrm{ CO}}_{2 (\mathrm{trapped})} }{\mathrm{h}}$$

where h = CO_2_ plume height (m), and, CO_2 (trapped)_ = the total amount of CO_2_ trapped by capillary forces (kg), which can be computed using the following equation:

12 $${\mathrm{CO}}_{2 (\mathrm{trapped})} = {\uprho }_{{\mathrm{CO}}_{2}}\sum_{1}^{\mathrm{n}}{\mathrm{V}}_{\mathrm{i}}{\upphi }_{\mathrm{i}}{\mathrm{S}}_{\mathrm{i}}$$

where,${\uprho }_{{\mathrm{CO}}_{2}}$ = density of supercritical CO_2_, taken to be 686.12 kg m^−3^, V_i_ = volume of the ith cell (m^3^), ϕ_i_ = porosity of the ith cell, S_i_ = CO_2_ saturation in the ith cell after plume stabilization, and n = number of grid cells in the model.

C_PMH_ helps in assessing if the same amount of CO_2_ is captured within a smaller reservoir volume. The amount of capillary trapping can be sensitive to the number of spatial dimensions due to reservoir heterogeneity. However, this variability can be safely ignored for this study all the scenarios represent nearly homogenous reservoirs.

### Predictor dominance analysis

Stepwise multi-linear regression was conducted to quantify the relative impact of six predictors of interest (Table S2) on the height, the width, and the amount of trapped CO_2_ per unit height of the plume for the 2D scenarios. The method estimates the importance of input variables by quantifying the change in R^2^ values resulting from a multi-variable regression model where one variable is excluded at each step. This allows the calculation of a marginal R^2^ value which is the difference between the value calculated with all six independent variables included, and the reduced R^2^ value obtained by omitting a predictor. These values were normalised to obtain the relative impact of each independent variable.

## Results and discussion

### Saturation distribution

**Figure 2:**
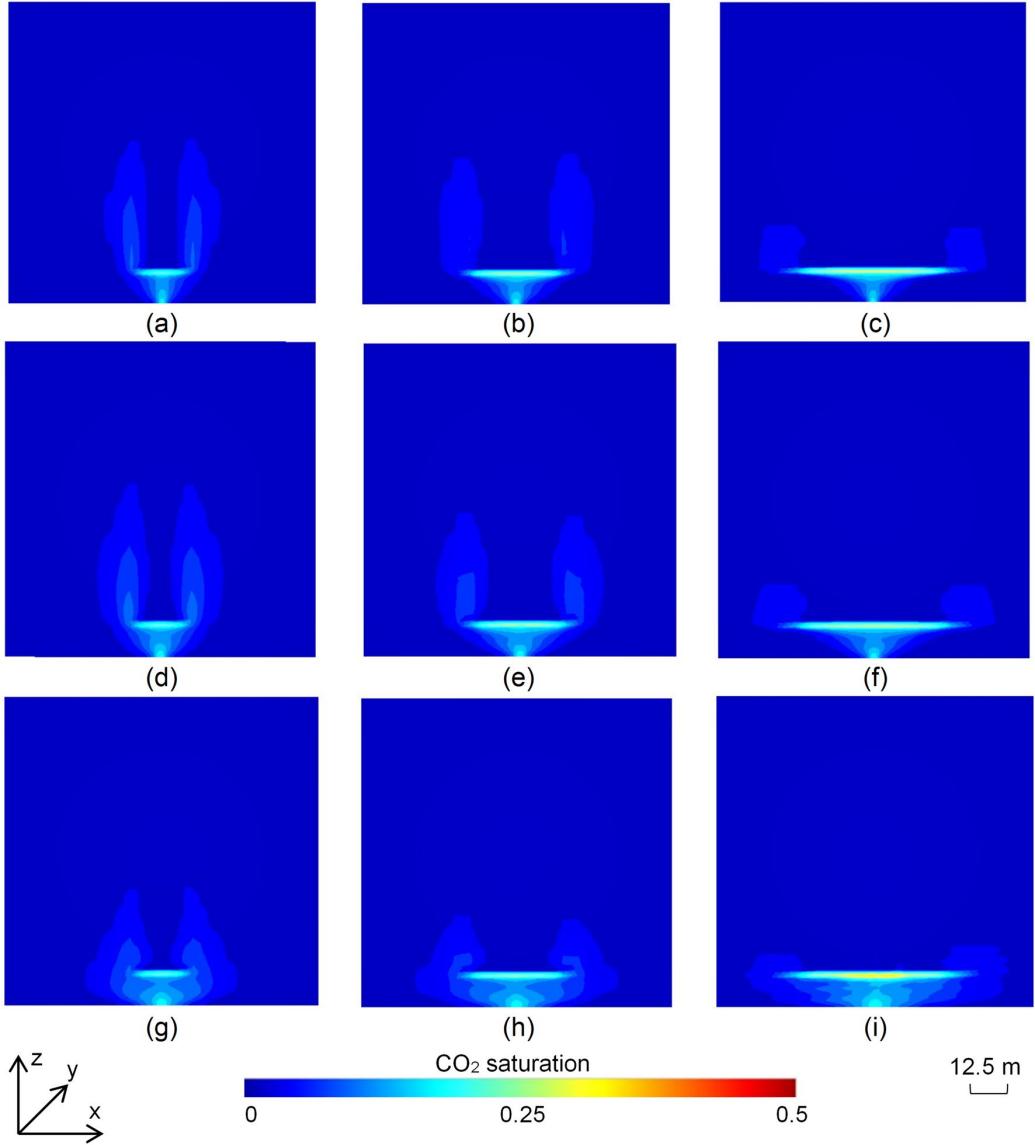
End-point (trapped) CO_2_ saturation distributions in the 2D scenarios with impermeable barrier with (**a**) k_mean_ (permeability) = 1697.57 mD and d_b_ (barrier diameter) = 15 m; (**b**) k_mean_ = 1206 mD and d_b_ = 30 m; (**c**) k_mean_ = 1175.73 mD and d_b_ = 50 m; (**d**) k_mean_ = 666.22 mD and d_b_ = 15 m; (**e**) k_mean_ = 747.44 mD and d_b_ = 30 m; (**f**) k_mean_ = 641.7 mD and d_b_ = 50 m; (**g**) k_mean_ = 220.31 mD and d_b_ = 15 m; (**h**) k_mean_ = 202.6 mD and d_b_ = 30 m; and (**i**) k_mean_ = 106.34 mD and d_b_ = 50 m.

The 2D scenarios were categorised into high (> 1000 mD) (Fig. 2a–c), moderate (500–1000 mD) (Fig. 2d–f) and low (100–500 mD) (Fig. 2g–i) permeability groups to determine the type of reservoir best suited for the use of a synthetic flow barrier. Nine of the 30 2D scenarios with impermeable barriers are illustrated in Fig. 2, demonstrating the effect of increasing barrier diameter for each permeability class. CO_2_ injected at the base of the models migrated to shallower depths driven by buoyancy, spreading laterally as it reached the impermeable flow barrier before continuing the upward migration on either side of the baffle (Fig. 2). Once the plume stabilised, the gas saturation ranged from 0.30 to 0.43 underneath the barrier where the gas was potentially mobile. Saturation values in the remaining plume, which was residually trapped on either side of the barrier, remained closer to the specified value of the maximum residual non-wetting phase saturation (Table S3), with less CO_2_ reaching the top of the plume. CO_2_ in the lower permeability scenarios (Fig. 2g–i) was generally restricted to deeper portions and reached a greater width compared to scenarios with moderate (Fig. 2d–f) and high permeability (Fig. 2a–c). While the total amount of residually trapped CO_2_ did not show much variation with increasing barrier diameters, the plume geometry was significantly affected, with extensive barriers creating wider plumes restricted to the deeper parts of the reservoir for each permeability class (Fig. 2). The detailed results for all 30 scenarios are shown in Fig. S3.

**Figure 3:**
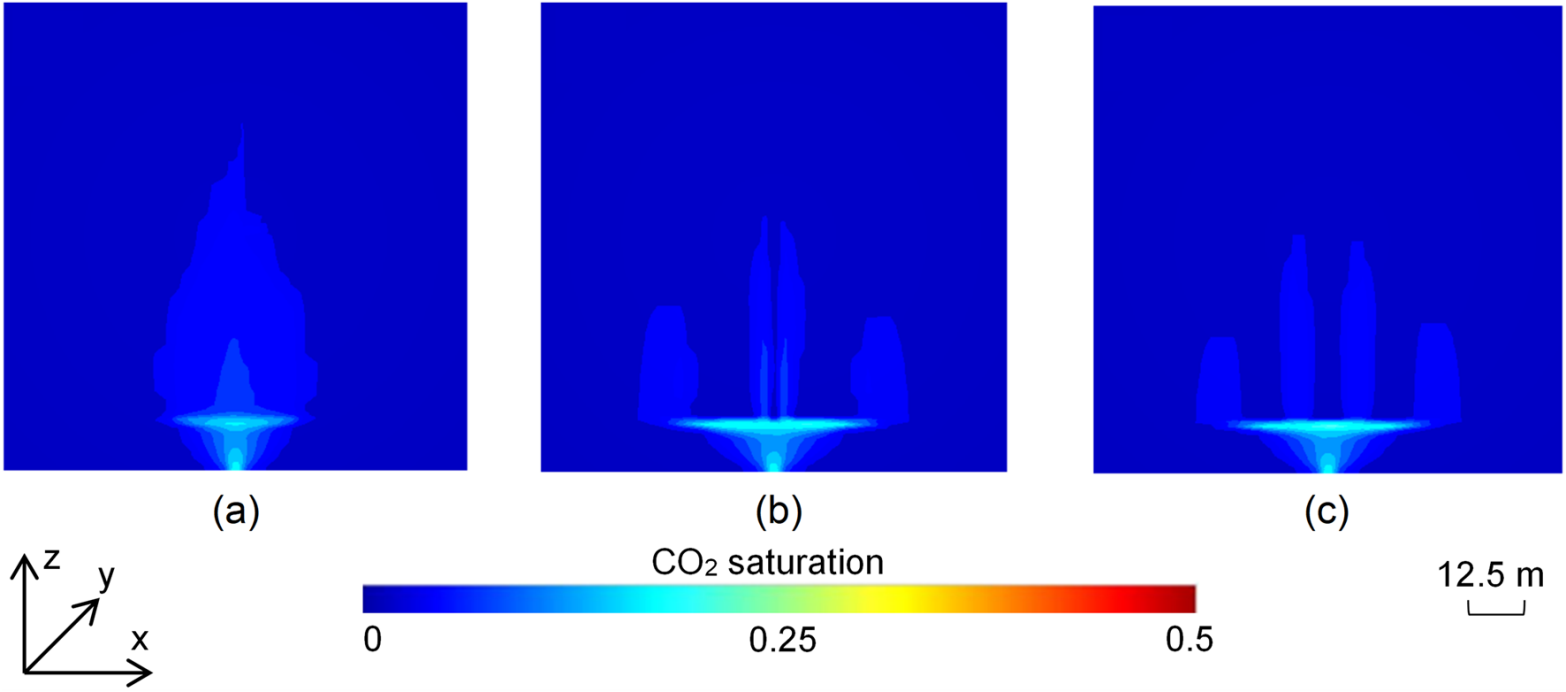
End-point (trapped) CO_2_ saturation distributions in the 2D scenarios comprising (**a**) a poor quality barrier with a permeability of 200 mD; (**b**) a partial barrier with two gaps 5 m apart; and (**c**) a partial barrier with two gaps 15 m apart.

The saturation distribution in the 2D scenarios with semi-permeable and partial barriers showed lower saturation build-up underneath the barrier as CO_2_ migrated through the barrier to shallower depths (Fig. 3). The scenario with a permeability of 200 mD for the barrier had a lower width of the plume as CO_2_ invaded through the barrier (Fig. 3a). However, the scenarios implementing gaps in the barrier showed a higher width of the plume underneath the barrier (Fig. 3b,c). These scenarios also showed that a certain volume of CO_2_ leaked through the gaps in the barrier. However, this amount is minor compared to CO_2_ volume trapped underneath the barrier. Additionally, the length of the gap in the barrier did not seem to have a significant impact on the width of the plume underneath the barrier and the volume of CO_2_ leaked though the barrier. The results suggest that discontinuously emplaced barriers could be more suitable than semi-permeable barriers towards enhancing the pore space utilization of the reservoir.

**Figure 4:**
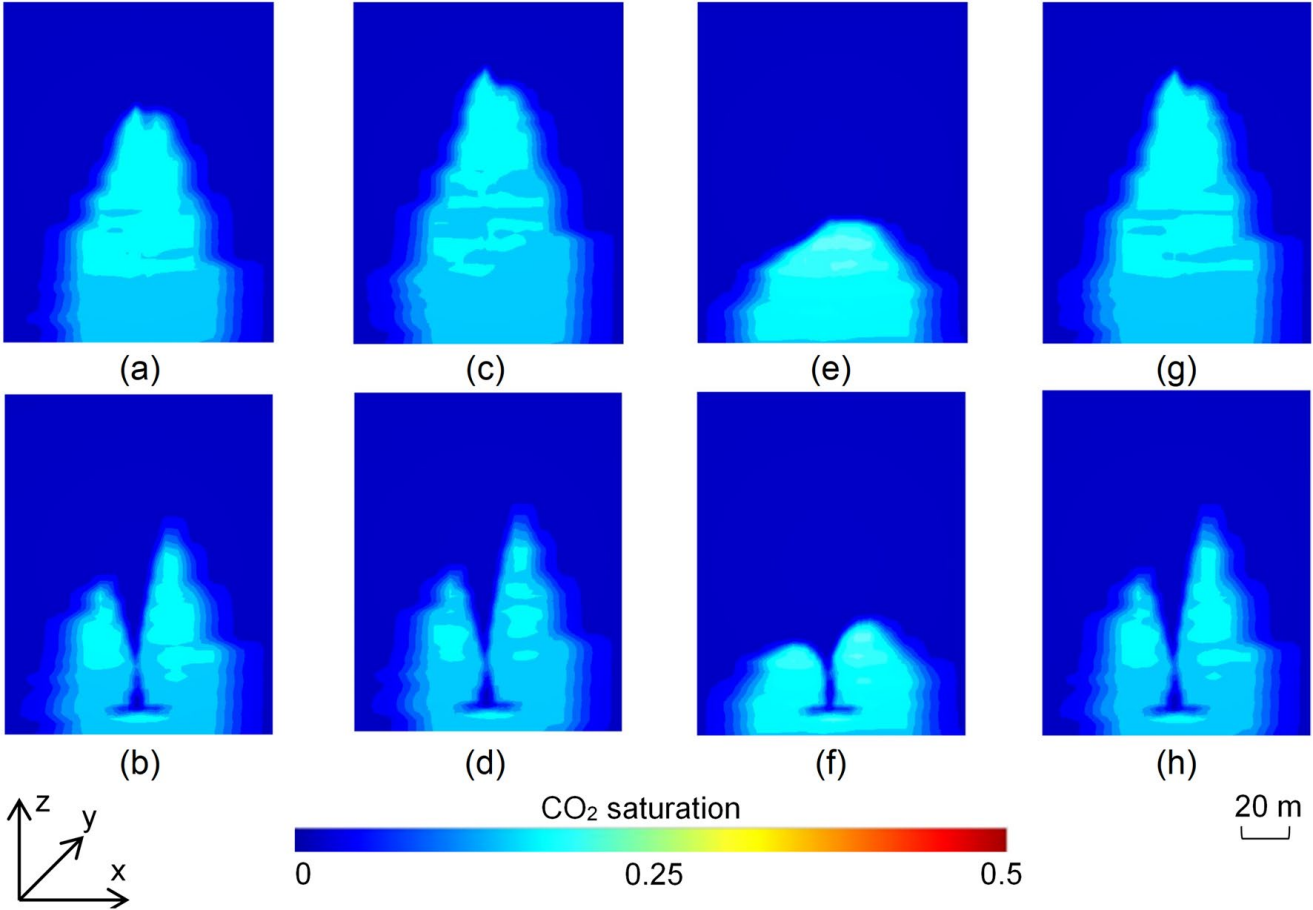
Turning-point CO_2_ saturation distributions in the 3D scenarios representing the deeper sections of the SW Hub site for (**a**) the reference case without barrier; (**b**) the reference case with barrier; (**c**) scenario with 1.4 × permeability of the reference case without barrier; (**d**) scenario with 1.4 × permeability of the reference case with barrier; (**e**) scenario with high anisotropy (k_v_/k_h_ = 0.1) without barrier; (**f**) scenario with high anisotropy (k_v_/k_h_ = 0.1) with barrier; (**g**) scenario with low anisotropy (k_v_/k_h_ = 0.8) without barrier; (**h**) scenario with low anisotropy (k_v_/k_h_ = 0.8) with barrier.

**Figure 5:**
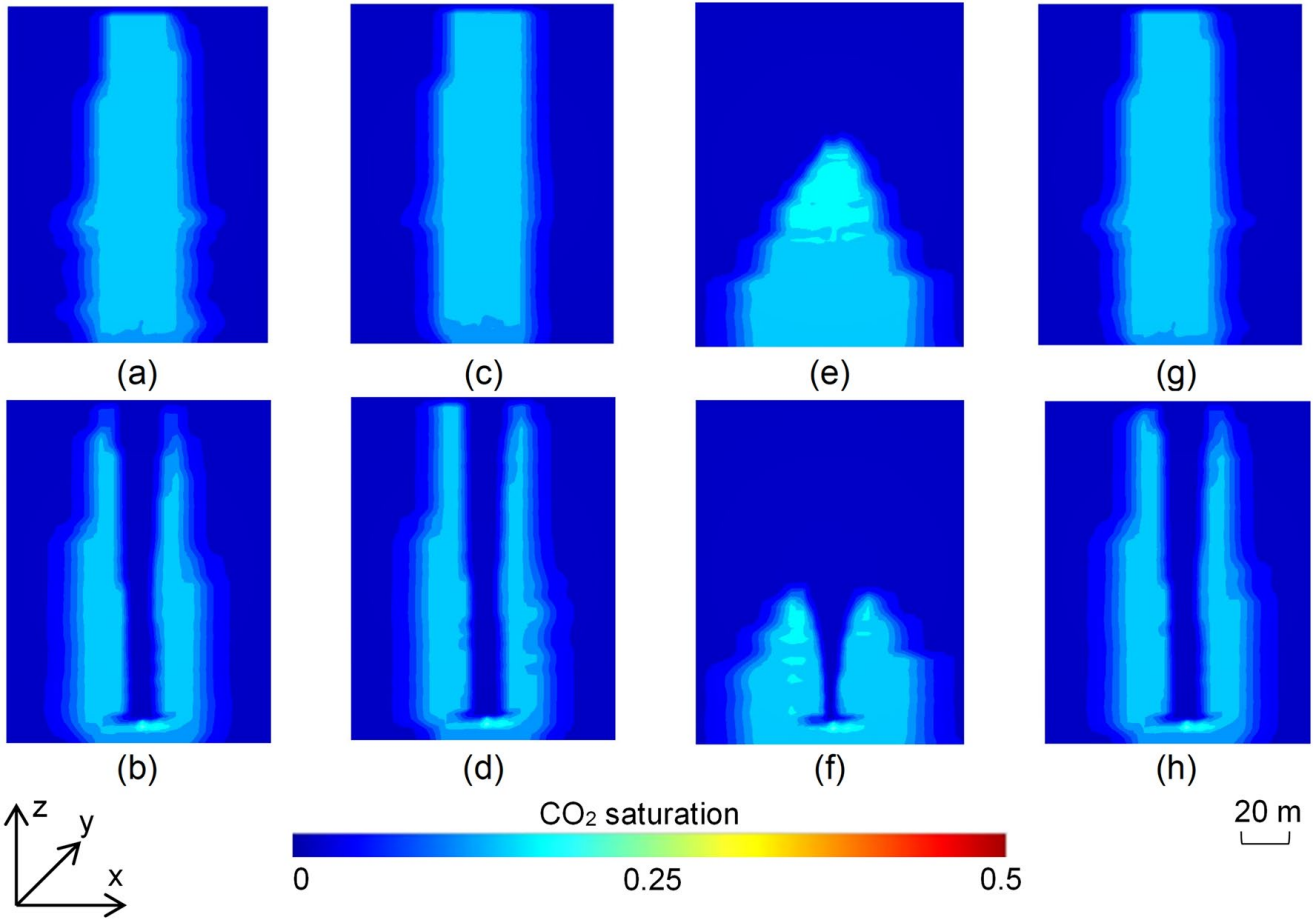
Turning-point CO_2_ saturation distributions in the 3D scenarios representing the shallower sections of the SW Hub site (with 10 × permeability of the scenarios representing deeper sections of the site) for (**a**) the reference case without barrier; (**b**) the reference case with barrier; (**c**) scenario with 1.4 × permeability of the reference case without barrier; (**d**) scenario with 1.4 × permeability of the reference case with barrier; (**e**) scenario with high anisotropy (k_v_/k_h_ = 0.1) without barrier; (**f**) scenario with high anisotropy (k_v_/k_h_ = 0.1) with barrier; (**g**) scenario with low anisotropy (k_v_/k_h_ = 0.8) without barrier; (**h**) scenario with low anisotropy (k_v_/k_h_ = 0.8) with barrier.

The saturation distributions in the 4 3D models capturing deeper sections of the SW Hub model (Fig. 4a,c,e,g) showed little variation except for the scenarios with high anisotropy which showed a smaller plume height for the same injected CO_2_ volume (Fig. 4e). The emplacement of the barrier clearly shows the restriction of the plume to deeper sections of the model (Fig. 4b,d,f,h). The 4 3D scenarios representing the shallower sections of the SW Hub site (Fig. 5a,c,e,g) showed a higher overall plume height compared to the corresponding scenarios representing the deeper section of the reservoir (Fig. 4a,c,e,g). The higher reservoir permeability also resulted in CO_2_ leakage from the top boundary of the model (Fig. 5a,c,g) though the emplacement of the barrier reduced this effect (Fig. 5b,d,h). This was, however, not the case for the scenario with high anisotropy (Fig. 5e,f) where the plume was restricted within the model domain.

### Impact of barrier emplacement on storage security and pore space utilization efficiency

**Figure 6:**
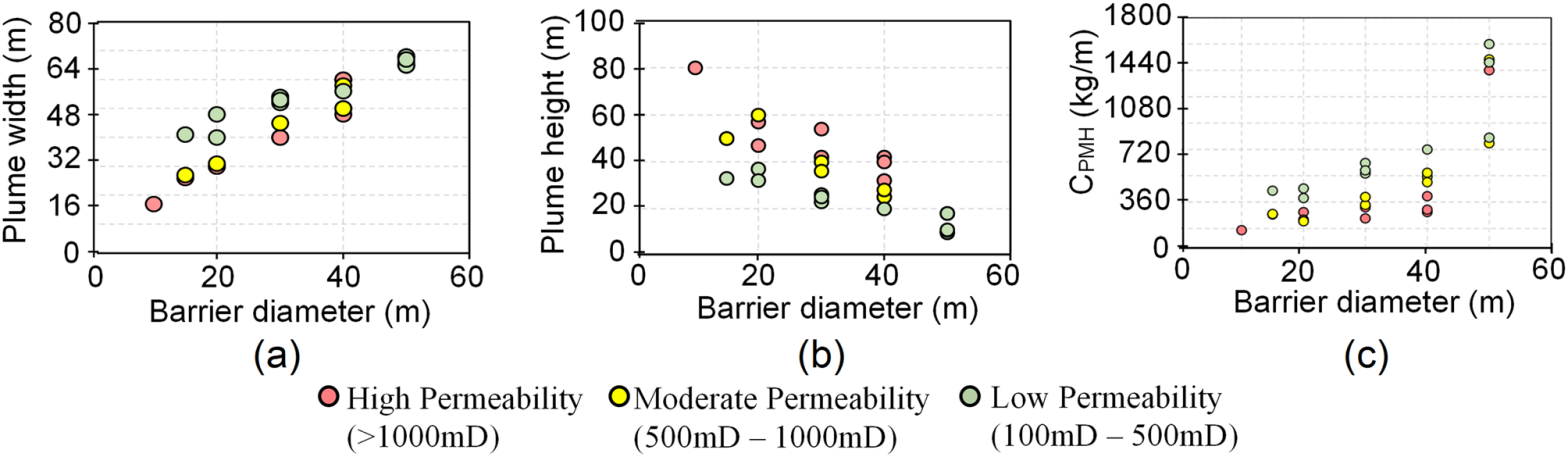
Relation between barrier diameter and (**a**) CO_2_ plume width; (**b**) CO_2_ plume height; (**c**) the amount of CO_2_ trapped per meter of plume height.

Though the height and the width of the plume varied throughout the 30 2D scenarios, there was no evident relationship between the plume geometry and intrinsic reservoir properties. However, the barrier width showed a considerable impact on CO_2_ plume geometry. Results indicate that a larger barrier diameter leads to increased width of the plume (Fig. 6a) with a corresponding decrease in height (Fig. 6b) for all ranges of reservoir permeability. This implies that wider barriers can potentially restrict the plume to greater reservoir depths, thereby contributing to enhanced storage security and pore space utilization as the same CO_2_ volume is trapped in a more laterally extensive manner. This is further confirmed by the dependence of CO_2_ trapped per metre of plume height (C_PMH_) on barrier diameter (Fig. 6c). C_PMH_ showed an increase with the increase in barrier diameter indicating that the emplacement of larger barriers leads to in an improved use of reservoir section further away from the injection well. A barrier size difference of 20 m in reservoir scenarios with similar properties resulted in a maximum increase of 39.97%, 48.13% and 61.43% in C_PMH_ for high, moderate and low permeability reservoirs, respectively (Fig. 6c). C_PMH_ was the highest in low permeability reservoirs, suggesting that pore space utilization efficiency is the most improved in tight reservoirs by the emplacement of a synthetic barrier. However, this could be due to a combined effect of increased barrier diameter and the low reservoir permeability both of which inhibit the buoyancy driven rise of CO_2_.

**Figure 7:**
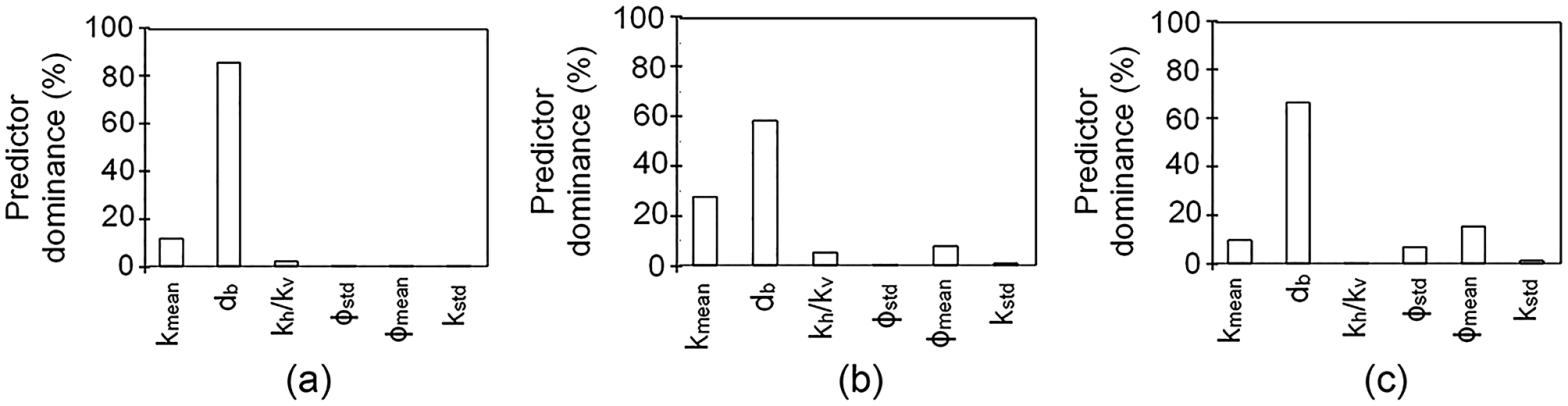
Dominance of different predictors (k_mean_ = mean permeability; d_b_ = barrier diameter; k_h_/k_v_ = ratio of horizontal to vertical permeability representing anisotropy; ϕ_std_ = standard deviation in porosity; ϕ_mean_ = mean porosity; k_std_ = standard deviation in permeability) on (**a**) CO_2_ plume width; (**b**) CO_2_ plume height; (**c**) the amount of CO_2_ trapped per meter of plume height.

The dominance of different reservoir and barrier properties, quantified using stepwise multi-linear regression analysis, revealed that the barrier diameter had the greatest significance with a relative impact of 85.97%, 58.53% and 66.72% on plume width, height, and C_PMH_, respectively (Fig. 7). The impact of mean permeability on the height and the width of the plume was 27.6% and 11.56%, respectively, while the other four input parameters each contributed 5% or less. Mean porosity had a relative impact of 15.4% on C_PMH_. These results indicate that the flow barrier is the primary factor influencing the geometry of the plume and the total amount of CO_2_ residually trapped per metre of plume height.

The 2D scenarios with semi-permeable and partial barriers showed that even such barriers are effective in reducing the buoyancy driven rise of the CO_2_ with a major proportion of the gas trapped as mobile fraction underneath the barriers (Fig. 3). The scenarios showed a higher pore space utilization vertically rather than laterally underneath the barrier, though CO_2_ saturation in the part of the plume above the barrier remained small in all the scenarios.

Results from the 3D simulations validated the observations made in the 2D scenarios. The emplacement of the barrier caused a reduction in the height of the plume. This was clearly evident in the scenarios capturing the shallower portions of the SW Hub reservoir where the plume was seen to leak out of the domain boundaries in all but the high anisotropy scenario (Fig. 5a,c,e,g). However, the presence of the barrier limited the leakage amount which was evident from the very low CO_2_ saturations around the top boundaries of the models (Fig. 5b,d,h). For the scenarios representative of the shallower sections of the site, the analysis revealed that there was a 24.8% reduction in the plume height in the high anisotropy scenario with a barrier. In the reference, high permeability and low anisotropy scenarios, there was an increase of 17%, 47% and 23.5%, respectively, in width with a barrier emplaced. C_PMH_ also showed an increase in all the 3D cases with the barrier emplaced. The reference case of the deeper SW Hub model section showed an increase of 9.66% in C_PMH_ with the barrier emplaced. However, there was only a 4% increase in the equivalent higher permeability reference case. The high anisotropy case with a barrier implemented in the shallower sections of the SW Hub site showed a 30% higher C_PMH_. In the other three cases of the shallower reservoir section, an increase in pore space utilization efficiency was also observed, though to a lesser extent at 4%, 5% and 7% for the reference, high permeability and low anisotropy scenarios, respectively.

The outcomes of this study suggest that the relationship between the size of the implemented barrier and the plume geometry and trapping primarily varies depending on the mean permeability of the reservoir. The synthetic fluid flow barrier emplacement technology can help in improving the security of CO_2_ trapping in reservoirs with an otherwise low capillary trapping efficiency. This is especially effective in reservoirs with high permeability (Fig. 5) which are likely to exist at shallower depths as part of coarse-grained depositional facies such as distributary channels. The storage security is not much affected in low permeability and highly anisotropic reservoirs which are more likely found in deep-seated tight reservoirs. This is because such properties already contribute towards restricting the rise of the plume. However, such reservoirs can greatly benefit in improving the pore space utilization efficiency by trapping higher CO_2_ amounts within smaller reservoir volumes (Fig. 6c).

## Conclusions

The presented study explores the impact of an artificially emplaced Si-gel fluid flow barrier on the storage security and the pore space utilization efficiency in unconventional CO_2_ storage reservoirs, especially the homogeneous open aquifers. The results indicate that the barrier can restrict vertical CO_2_ plume migration above the injection location by enhancing the lateral migration of the plume. This leads to plume containment in deeper regions of the reservoir which improves storage security. Additionally, higher volume of CO_2_ is trapped underneath the barrier which increases the pore space utilization efficiency. The containment of CO_2_ in this part of the reservoir is enhanced by capillary forces which restrict mobile CO_2_ underneath the barrier. The semi-permeable and partial Si-gel barriers, emplaced from a poor precipitation of the silica polymer or from a sequential injection of silica and water, also contribute towards enhancing the security and the efficiency of trapping though the enhanced pore space utilization occurs vertically rather than laterally underneath the barrier. Overall, the findings of this study suggest that the artificial emplacement of a Si-gel barrier is particularly useful in reservoirs where capillary trapping is the primary CO_2_ storage mechanism and leads to a higher degree of pore space utilization, especially in relatively homogeneous reservoirs with high permeability.

## Supplementary Information

Supplementary Information 1.

Supplementary Information 2.
