# Supplementary material for: The feasibility of enhanced pore space utilization in CO2 storage reservoirs using an artificially emplaced Si-gel flow barrier
Source: Sci Rep. 2023 Jun 8;13:9334. doi: 10.1038/s41598-023-36349-0 (PMC10250531; doi:10.1038/s41598-023-36349-0)
Supplement: Supplementary file 1 — Supplementary Information 1. [file 41598_2023_36349_MOESM1_ESM.pdf]

Supplementary data

The supporting figures and tables are provided below.

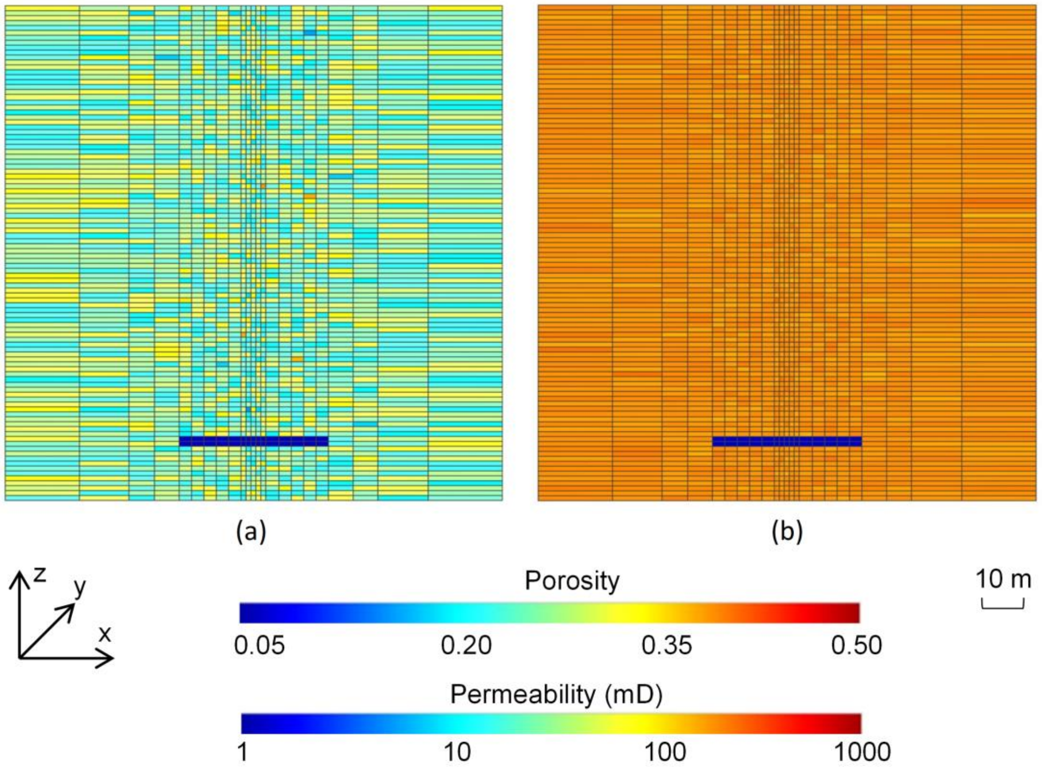

Figure S1. A representative 2D geostatic model for scenario 4 with a) porosity (mean =0.27, standard deviation = 0.03); and b) permeability (mean = 1570 mD, standard deviation = 17.44 mD) distributions. The barrier, which is 30 m in this scenario, has a porosity of 0.05 and permeability of 1 mD. The domain has a length and a height of 100m each. The grid cells measured 1m vertically while their sizes varied between 1m and 15m horizontally.

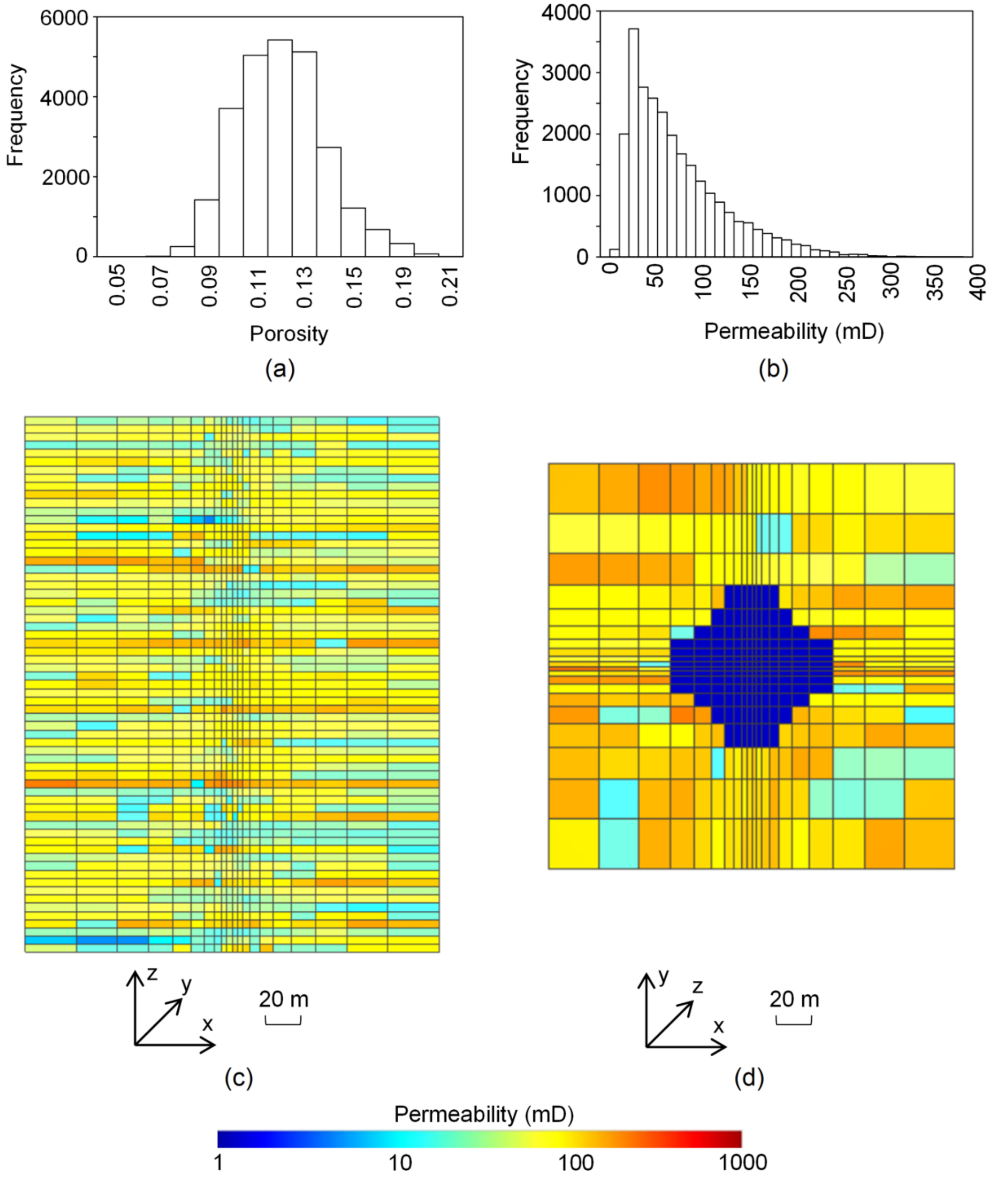

Figure S2. (a) Porosity; and (b) permeability values from the available SW Hub data used to populate the domains of the 3D reservoir models in this study. Permeability distribution in (c) the front view; and (d) the layer with the Si-gel barrier in the plan view in the reference case 3D scenario.

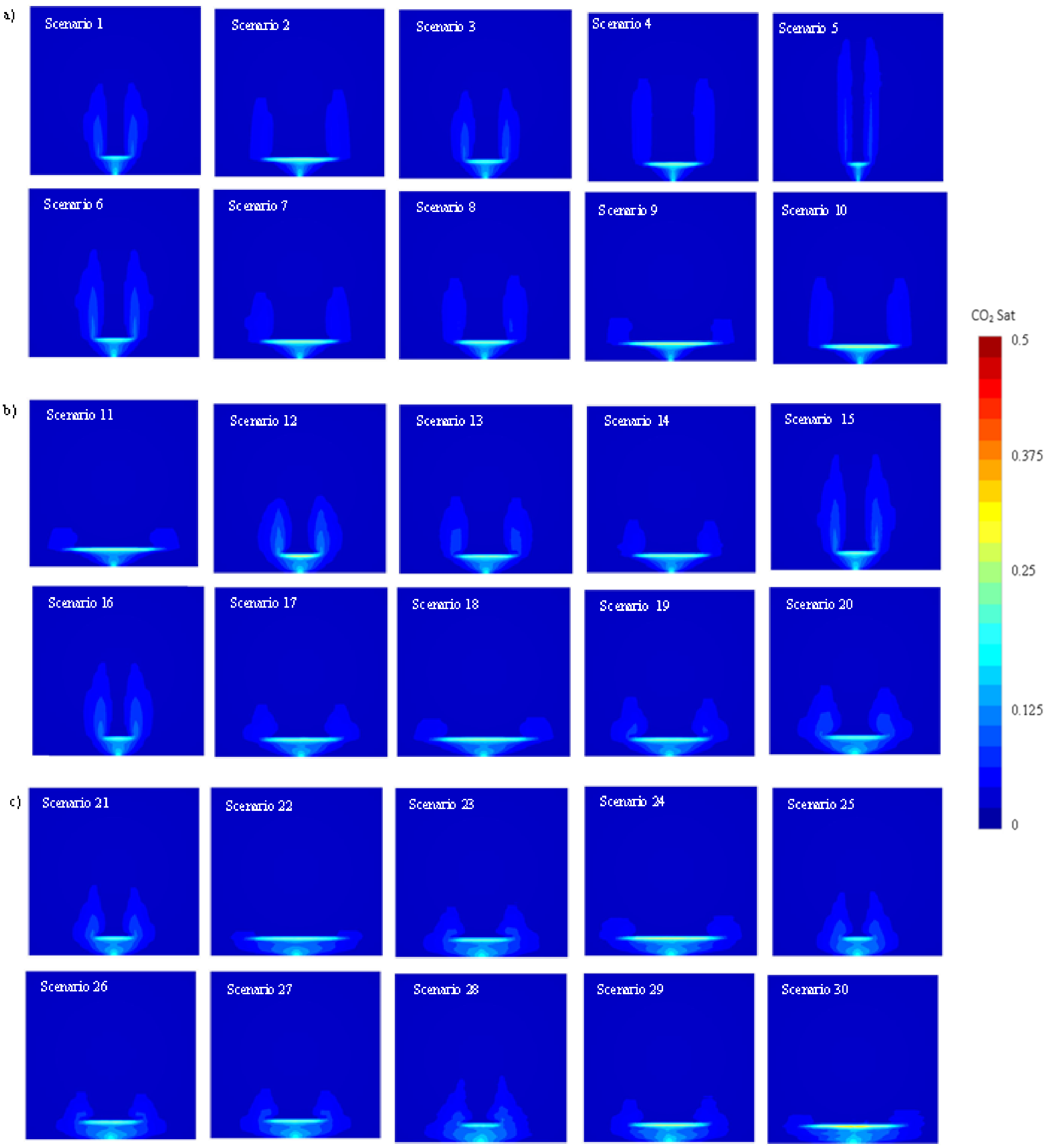

Figure S3. CO_2 saturation distribution after 100 days of 2D simulation in a) high permeability (>1000mD); b) moderate permeability (500 – 1000 mD); and c) low permeability (<500 mD) scenarios, each with varying barrier diameters and reservoir properties specified in Table S1.

Table S1. Barrier Diameter, anisotropy ratio (k_v/k_h), porosity mean and standard deviation, and permeability mean and standard deviation for each 2D scenario.

Scenario ID	Barrier Diameter	k_v/k_h	ϕ mean (fraction)	ϕ Std (fraction)	k_p mean (mD)	k_p std (mD)
**1**	15	0.35	0.35	0.03	1697.57	6.16
**2**	40	0.39	0.28	0.05	1642.88	24.15
**3**	20	0.34	0.34	0.05	1630.71	39.29
**4**	30	0.46	0.27	0.03	1570.44	17.14
**5**	10	0.77	0.33	0.05	1423.51	45.05
**6**	20	0.35	0.26	0.01	1418.85	28.64
**7**	40	0.39	0.31	0.05	1329.61	27.86
**8**	30	0.45	0.29	0.02	1206	37.23
**9**	50	0.56	0.34	0.03	1175.73	43.33
**10**	40	0.55	0.27	0.02	1077.75	35.2
**11**	50	0.43	0.34	0.02	996.18	22.45
**12**	40	0.37	0.28	0.02	887.07	47.88
**13**	30	0.52	0.27	0.02	747.44	42.65
**14**	50	0.6	0.35	0.02	747.02	33.89
**15**	20	0.63	0.23	0.03	715.03	40.71
**16**	15	0.58	0.28	0.02	666.22	5.93
**17**	40	0.44	0.31	0.02	655.03	48.27
**18**	50	0.42	0.3	0.03	641.7	25.72
**19**	40	0.45	0.27	0.05	545.81	24.67
**20**	30	0.38	0.23	0.04	502.33	43
**21**	20	0.55	0.32	0.02	438.74	16.61
**22**	50	0.39	0.33	0.01	360.69	37.26
**23**	30	0.39	0.28	0.03	258.41	44.99
**24**	40	0.65	0.25	0.04	236.09	42.88
**25**	15	0.62	0.34	0.01	220.31	5.18
**26**	30	0.45	0.31	0.04	216.83	22.51
**27**	30	0.55	0.29	0.02	202.6	10.38
**28**	20	0.39	0.28	0.04	171.07	38.41
**29**	40	0.8	0.28	0.03	125.55	27.31
**30**	50	0.74	0.26	0.02	106.34	40.24

Table S2. Range of different reservoir and barrier properties used in the 30 2D models.

Property	Barrier diameter (m)	Anisotropy (k_v/k_h)	Mean porosity (fraction)	Porosity std. deviation (fraction)	Mean permeability (mD)	Permeability std. deviation (mD)
**Range**	15–50	0.333–1	0.25–0.35	0.01–0.05	100–2000	5.18–48.27

Table S3. Parameters used in the saturation functions for the 2D and the 3D models.

Scenario	Property	Reservoir rock	Barrier
2D	P_o (kPa)	1	10
2D	m	0.70	0.41
2D	$s_{nw,max}$	0.19	0.50
2D	$s_{w,irr}$	0.49	0.27
3D	P_o (kPa)	8	1,400
3D	m	0.457	0.333
3D	$s_{nw,max}$	0.12	0.50
3D	$s_{w,irr}$	0.37	0.27
